# EnContact: predicting enhancer-enhancer contacts using sequence-based deep learning model

**DOI:** 10.7717/peerj.7657

**Published:** 2019-09-13

**Authors:** Mingxin Gan, Wenran Li, Rui Jiang

**Affiliations:** 1 Donlinks School of Economics and Management, University of Science and Technology Beijing, Beijing, China; 2 MOE Key Laboratory of Bioinformatics; Bioinformatics Division and Center for Synthetic and Systems Biology, BNRist; Department of Automation, Tsinghua University, Beijing, China; 3 Department of Statistics, Stanford University, Stanford, CA, USA; 4 Department of Biomedical Data Science, Stanford University, Stanford, CA, USA

**Keywords:** Deep learning, HiChIP data, Attention-based RNN, Hub enhancers, Enhancer-enhancer contacts

## Abstract

Chromatin contacts between regulatory elements are of crucial importance for the interpretation of transcriptional regulation and the understanding of disease mechanisms. However, existing computational methods mainly focus on the prediction of interactions between enhancers and promoters, leaving enhancer-enhancer (E-E) interactions not well explored. In this work, we develop a novel deep learning approach, named Enhancer-enhancer contacts prediction (EnContact), to predict E-E contacts using genomic sequences as input. We statistically demonstrated the predicting ability of EnContact using training sets and testing sets derived from HiChIP data of seven cell lines. We also show that our model significantly outperforms other baseline methods. Besides, our model identifies finer-mapping E-E interactions from region-based chromatin contacts, where each region contains several enhancers. In addition, we identify a class of hub enhancers using the predicted E-E interactions and find that hub enhancers tend to be active across cell lines. We summarize that our EnContact model is capable of predicting E-E interactions using features automatically learned from genomic sequences.

## Introduction

Chromatin contacts between regulatory elements are widely studied to interpret the regulation relationship of transcriptome and to understand the regulatory mechanism of complex diseases. Chromosome conformation capture (3C)-based methods, including 4C and 5C, have been developed to detect physical contacts on a local scale (Dekker et al., 2002; Simonis et al., 2006; Dostie et al., 2006). Chromatin Interaction Analysis by Paired-End Tag Sequencing (ChIA-PET) captures chromatin interactions related to a protein of interest (Fullwood et al., 2009). Recently, Hi-C, Capture Hi-C, and HiChIP techniques allow genome-wide detection of interactions between all possible pairs of regions (Rao et al., 2014; Mifsud et al., 2015; Mumbach et al., 2016), which provides the comprehensive landscape of three-dimension chromatin structure. However, all of these techniques require an extremely deep sequencing depth to achieve high resolution, which can hardly be applied to a large number of cell lines. Therefore, computational approaches are needed to help with the identification of finer-mapping interactions.

In the past 5 years, a series of methods have been developed to predict promoter-related interactions (Roy et al., 2015; Whalen, Truty & Pollard, 2016; Li, Wong & Jiang, 2019; Cao et al., 2017; Schreiber et al., 2018; Singh et al., 2016). Roy et al. (2015) implemented regulatory interaction prediction for promoters and long-range enhancers which integrates published 3C data sets with a minimal set of regulatory genomic data sets to predict enhancer-promoter interactions in a cell line-specific manner. Whalen, Truty & Pollard (2016) proposed a computational method, TargetFinder, to predict promoter-enhancer interactions from diverse features along the genome. With the understanding that there exist enhancer-like promoters which can regulate distal genes (Diao et al., 2017; Gasperini et al., 2017), Li, Wong & Jiang (2019) developed a deep learning model, DeepTACT, which pays equal attention to the prediction of promoter-enhancer interactions and promoter-promoter interactions. However, all of these methods only focused on the prediction of promoter-enhancer interactions and promoter-promoter interactions.

Over the last decade, studies have demonstrated the importance of enhancer-enhancer (E-E) interactions in the interpretation of gene regulation (Ghavi-Helm et al., 2014; Ing-Simmons et al., 2015; Kumasaka, Knights & Gaffney, 2019; Mumbach et al., 2017). Ghavi-Helm et al. (2014) generated a high-resolution map of enhancer 3D contacts during Drosophila embryogenesis and found each enhancer contacts multiple enhancers and promoters with similar expression, suggesting a role of co-regulation of enhancers. Ing-Simmons et al. (2015) reported that ∼50% of deregulated genes in mouse thymocytes reside in the vicinity of enhancer elements, suggesting that gene expression was regulated through E-E interactions. Kumasaka, Knights & Gaffney (2019) detected frequent long-range interactions between enhancers, which were further used to interpret gene regulation and disease causality. Although studies have shown that E-E loops are closely related to gene expression by co-regulation, to the best of our knowledge, hardly any computational approach is specifically designed to provide a powerful prediction of E-E contacts.

In computational biology filed, genomic sequence information is widely used to extract motif-like patterns to predict regulatory elements (e.g., enhancers) using various statistical models (Le et al., 2019; Kleftogiannis, Ashoor & Bajic, 2018). Recently, deep learning has shown impressive performance in pattern recognition and imaging field (Shen et al., 2017; Wang, Sun & Wang, 2017). By formatting genomic sequences and other epigenomic information into numeric vectors or matrix, deep learning models have achieved great success in many biological problems such as the identification of transcription factor (TF) binding sites, the recognition of regulatory regions, and the prediction of interacting pairs (Li, Wong & Jiang, 2019; Kelley, Snoek & Rinn, 2016; Alipanahi et al., 2015; Park & Kellis, 2015; Le, Ho & Ou, 2017, 2018; Le & Nguyen 2019). These applications demonstrate the ability of deep learning models in capturing useful genomic features and accurately predicting biological signals. This inspires us to design a deep learning model to predict E-E interactions by learning motif-like feature patterns from genomic sequences using deep neuron networks.

In this paper, we develop a deep learning approach, named Enhancer-enhancer contacts prediction (EnContact), to predict E-E contacts only using genomic sequences. Through a series of comprehensive experiments in seven cell lines, we demonstrate that EnContact is able to learn context-specific features from genomic sequences and to predict E-E interactions accurately. When applying the context-specific models to HiChIP data, EnContact identifies finer-mapping E-E interactions from region-based chromatin contacts, where each region contains several enhancers. In addition, EnContact identifies a class of hub enhancers which are active across different cell lines. In summary, EnContact achieves much better performance compared with traditional machine learning methods and is capable of identifying E-E interactions from HiChIP data.

## Materials and Methods

### Collection and processing of datasets

We collected chromatin contact matrix of seven cell lines (i.e., GM, K562, HCASMC, MyLa, Naïve, Th17, and Treg) from HiChIP data of Mumbach et al. (2017). Permissive enhancers were downloaded from FANTOM5 (Andersson et al., 2014) and then uniformly extended to two kb based on their middle sites for more information in their surrounding genomic sequence. In each cell line, we converted the contact matrix into chromatin interactions and annotated each bait-level interaction with permissive enhancers, resulting in a list of enhancer-related interactions. Then, those enhancer-related interactions were divided into two subsets: a “1v1” set which contains interaction pairs with only one enhancer in each end; a “mvm” set which consists of interaction pairs with more than one enhancer in either end or both ends (Table 1).

**Table 1:** Number of enhancer-enhancer interactions collected from HiChIP.

Cell type	Acronym	1v1	mvm
GM12878	GM	143,810	144,380
K562	K562	158,058	142,131
Human coronary artery smooth muscle	HCASMC	110,078	114,758
CD4+ T cell leukemia	MyLa	124,858	123,163
Naïve CD4+ T cells	Naive	128,450	146,308
T helper 17 cells	Th17	145,706	178,600
T regulatory cells	Treg	112,392	125,625
Total	–	923,352	974,965

Chromatin interaction analysis by paired-end tag sequencing data of different cell lines were collected from Tang et al. (2015) and processed using a standard tool ChIA-PET2 (Li et al., 2017) with default settings, yielding 194,467 fragment-level interactions at a *q* value threshold 0.05. Then, we annotated these fragment-level interactions with enhancers using bedtools (Quinlan & Hall, 2010), resulting in a list of 37,894 E-E interactions as a validation set. DNase-seq experiments of above seven cell lines and ChIP-seq experiments of four histone marks (i.e., H3K4me3, H3K27ac, H3K4me2, H3K9ac) and 579 TFs were downloaded from ENCODE (The ENCODE Project Consortium, 2004). Detailed experimental information is shown in Tables S1–S3.

### Structure of EnContact

The structure of EnContact can be divided into three parts: two convolution neuron networks (CNN) and a recurrent neural network (RNN). In each CNN, a convolution layer is used to learn motif-like patterns from genomic sequences, together with a rectifier operation (rectified linear unit (ReLU)) to propagate positive outputs and eliminate negative outputs. The convolution process can be devoted as

(1) $${\rm{Conv}}{\left( X \right)_{ij}} = {\rm{ReLU}}\left( {\mathop \sum \limits_{m\ =\ 1}^M \mathop \sum \limits_{n\ =\ 1}^N {w_{m,n}}{x_{i\ +\ m - 1,\ j\ +\ n - 1}}} \right),$$

where *X* is the one-hot encoding representation of sequences, $i = 1,2, \cdots ,L - M + 1;$ *j* = *C* − *N* + 1. Here, *L* is the length of an enhancer (i.e., *L* = 2,000), *C* the number of nucleotides (i.e., *C* = 4). $$W = {\left( {{w_{mn}}} \right)_{M\  \times\ N}}$$ is the weight matrix of a convolution kernel; $M \times N$ the size of the kernel. In our case, *N* is set to be 4, thus *j* is a constant 0. The activation function is the ReLU, defined as

(2) $${\rm{ReLU}}\left( z \right) = {\rm{max}}\left\{ {0,z} \right\}.$$

Next, max-pooling layers are used to reduce dimensions and help extract higher-level features. The pooling process can be devoted as

(3) $${\rm{MaxPooling}}\left( X \right) = \max \left\{ {{x_{ij}},{x_{i\ +\ 1,j}}, \cdots ,{x_{i\ +\ W,j}}} \right\},$$

where *W* is the size of pooling window.

Then, features learned by the above CNNs are concatenated using a merging layer, followed by a bidirectional long-short-term memory (BLSTM) layer to further learn the context features from pooled sequence patterns. As a typical representation of RNNs, BLSTM is widely used for its ability in capturing dependencies of sequences by accessing long-range context (Graves, Jaitly & Mohamed, 2013). To help BLSTM to pay more attention to specific sequence patterns, an attention layer is adopted in the integration module, following the BLSTM layer. The simplification of attention mechanism can be formulated as

(4) $${\alpha _t} = {{{\rm{exp}}\left\{ {f\left( {{h_t}} \right)} \right\}} \over {\sum\nolimits_{K = 1}^T {\exp \left\{ {f\left( {{h_k}} \right)} \right\}} }}$$

where ${\rm{}}f\left( \cdot \right) = {\rm{tanh}}\left( \cdot \right)$ can be considered as a learnable function depending on hidden layer *h_t_* at time step *t*, which measures scalar importance for *h*_*t*_. α*_t_* is the weight computed at each time step *t* for each state ${h_t},t = \left( {1,2, \cdots ,T} \right)$; *T* the number of time steps determined by the BLSTM layer. Given hidden states *h*_*t*_, attention layer computes an adaptive weighted average of hidden states, θ, devoted as

(5) $${\rm{\theta }} = \sum\limits_{t = 1}^T {{\alpha _t}{h_t}}.$$

The final layer of EnContact is a dense layer which is actually an array of hidden units with the ReLU activations feeding into a logistic regression (LR) unit that predicts the probability of interacting. In addition, we adopt batch normalization layers to accelerate the training process and dropout layers to avoid overfitting. Details for the parameters used in the deep learning model are described in Table S4. We implemented the EnContact model using Keras 1.2.0 (Bastien et al., 2012) on a Linux server. All experiments were carried out with 4 Nvidia K80 GPUs which significantly accelerated the training process than CPUs.

### Generating negative cases based on the distance distribution

We generate negative cases based on the distance distribution of positive cases. Following existing literature (Whalen, Truty & Pollard, 2016), we first divide the distances between positive interaction pairs into five bins, guaranteeing each bin has an equal number of positive samples. Then, we generate negative cases within each distance bin, making sure that the number of negative cases in each bin is the same as that of positive cases.

### Baseline methods

We use four baseline methods for comparison, including three typical classification models, SVM (Wu, Lin & Weng, 2004), LR (Hosmer, Lemeshow & Sturdivant, 2013), and random forest (RF) (Liaw & Wiener, 2002), and a deep learning model SPEID (Singh et al., 2016). For the typical classification models, to convert nucleotide-based information into numeric vectors, we extract *k*-mer features from genomic sequences using the following strategy. First, we list all combinations of four types of nucleotides (A, T, C, G) in *k* sites, resulting in 4*^k^* motif-like patterns. Then, for the sequence of a given enhancer, we count the occurrence frequency of each *k*-mer pattern. Thus, we derive a feature vector consisting of the frequency of 4*^k^* motif-like fragments for each enhancer. Since our goal is to predict the interaction of two enhancers, we connect the feature vectors of these two enhancers as the input for baseline methods.

We accomplish the classification process of SVM, LR, and RF using Scikit-learn package (Pedregosa et al., 2011) in Python. We downloaded the source code of SPEID from https://github.com/ma-compbio/SPEID and ran the model followed its instruction. Considering SPEID was designed to predict enhancer-promoter contacts, we substituted promoter-enhancer sequences to E-E sequences as the input for SPEID. The input samples and features are totally the same for SPEID and EnContact. To ensure a fair comparison, we provide the same training sets and testing sets in seven cell lines for baseline methods and our EnContact model.

### Motif analysis

To convert the weights of convolution kernels learned by EnContact into probabilistic position weight matrix (PWM), we first calculate the activation scores of kernels for a given input (i.e., the sequence of an enhancer), as

(6) $${S_i} = \mathop \sum \limits_{m\ =\ 1}^M \mathop \sum \limits_{n\ =\ 1}^N {w_{m,n}}{x_{i\ +\ m - 1,n - 1}},$$

where *S_i_* is the activation score of the *i*th nucleotide of the input sequence. Then, we define a position as activated if its activation score is larger than half of the maximum value of the whole sequence, formulated as

(7) $$\left\{ \matrix{ {S_i} > 0.5*{\rm{MAXS}} \hfill \cr {\rm{MAXS}} = \max \left\{ {{S_i}|1 \le i \le L} \right\} \hfill \cr} \right.\hskip -3pt,$$

where *L* is the length of the sequence; MAXS the maximum value of activation scores.

Then, we count the nucleotide occurrences of activated positions and format them into PWMs. After that, we match the resulting PWMs to known motifs derived from JASPAR database (Mathelier et al., 2016), which contains 1,082 motifs of 1,072 human TFs, using the tool TOMTOM v4.12.0 (Gupta et al., 2007) with a threshold of false discovery rate (FDR) *q* value < 0.1.

### Definition of co-opening degree between two enhancers

We define the co-opening degree of two enhancers as the absolute value of Pearson’s correlation coefficient of their openness scores. Here, the openness score of each enhancer is defined as follow. Suppose the number of replicates in a given cell line is *R*, the length of the regulatory element *L*, then the DNase signal of each regulatory element can be represented as *O^R^* ^×^ *^L^*, where ${O_{rl}}\; \left( {r = 1,2, \ldots ,R;\; l = 1,2, \ldots ,L} \right)\;$ is the chromatin accessibility score at each genomic site, defined as

(8) $${O_{rl}} = {\rm{}}{{{N_{rl}}} \over {{M_{rl}}/W}},$$

where *N* is the number of reads falling at each genomic site; *M* the number of reads falling into a background window of length *W* (say, one Mb) surrounding this site. This fold change value ${O_{rl}}$ is designed to remove the influence of sequencing depth according to Li et al. (2017).

Then, for each enhancer, we obtain a vector of openness scores by averaging *O^R^* ^×^ *^L^* across replicates *R*. Finally, the co-opening degree of two regulatory elements is defined as the absolute value of Pearson’s correlation coefficient of two vectors of openness scores. If the *P*-value of a Pearson’s correlation coefficient of an interaction pair is significant (i.e., *P*-value > 0.05), we consider this E-E pair as co-opening.

### Activity of hub enhancers

For each peak *i* in experiment *j*, we define an activity score, PAS*_ij_*, by calculating the fold change between the number of reads falling into this peak and the number of reads falling into a background region surrounding the peak, formulated as

(9) $${\rm{PA}}{{\rm{S}}_{ij}} = {\rm{}}{{{N_{ij}}/{P_{ij}}} \over {{M_{ij}}/W}},$$

where *W* is the length of background window, defaulted as one Mb; *P_ij_* the length of peak *i* in experiment *j*; *N_ij_* the number of reads falling into peak *i* in experiment *j*; *M_ij_* the number of reads falling into the background region of peak *i* in experiment *j*.

Then, we define the activity score of an enhancer as the maximum activity score of peaks overlapping with this enhancer,

(10) $${\rm{EA}}{{\rm{S}}_{ij}} = \max \left\{ {{\rm{PA}}{{\rm{S}}_{kj}}{\rm{|}}k \in {S_i}} \right\},$$

where *S_i_* is the set of peaks overlapping with enhancer *i*. Finally, we consider an enhancer as active when its activity score is greater than 1. For each hub enhancer or non-hub enhancer, we count the number of experiments where it is active to assess its activity across cell lines.

## Results

### Design of EnContact model and training strategy

**Figure 1:**
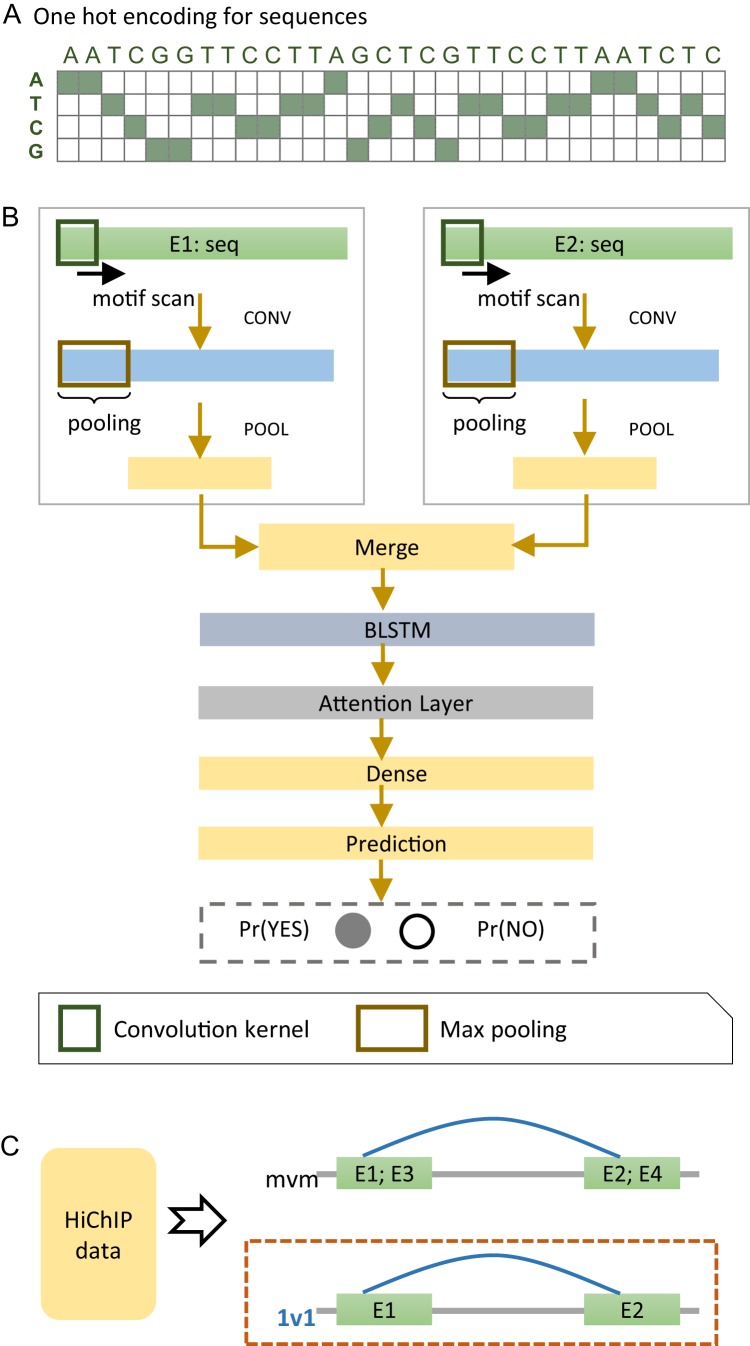
The EnContact method. (A) One-hot encoded sequence matrix. (B) Schematic illustration of the deep neural network architecture in EnContact. See “Methods” for details. (C) Region-based interactions in HiChIP data are divided into two sets: one set consists of interacting regions with only one enhancer in each region (1v1); the other set contains interacting regions where each region contains more than one enhancer (mvm).

We developed a deep learning model, named EnContact, to identify E-E interactions using features learned from genomic sequences. As shown in Fig. 1A, we adopted a one-hot encoding strategy to convert the sequence of an enhancer into a four-dimensional matrix, where each genomic site has a four-element vector with the nucleotide bit set to be 1. For a given E-E pair, EnContact learns patterns from the encoded sequences of two enhancers using two separate convolutional neuron networks. Then, an attention-based RNN was applied to extract high-level features from the concatenation of patterns learned by CNNs. Finally, EnContact predicts the interacting probability of the given two enhancers using a LR layer (Fig. 1B). Details of EnContact model are shown in Methods.

To construct context-specific training data for EnContact, we developed the following strategy. First, we collected the chromatin interactions of seven cell lines from the HiChIP data of Mumbach et al. (2017). Then, we annotated those interactions with permissive enhancers derived from FANTOM5 (Andersson et al., 2014) to obtain E-E interactions. As shown in Fig. 1C, the E-E interactions are further divided into two subsets: one subset consists of interactions that have only one enhancer located at each end (1v1); the other subset contains interactions which have more than one enhancer in either end or both ends (mvm). Thus, we derived an average of 131,907 interacting E-E pairs for “1v1” subsets and an average of 1,023,134 interacting pairs for "mvm" subsets. The numbers of E-E interactions collected from the HiChIP data of seven cell lines are shown in Table 1. Next, we use the unambiguous E-E interactions in “1v1” subsets to construct positive cases for model training and cross validation. After a context-specific model is trained using “1v1” subset, we then apply the model to identify true E-E interactions from ambiguous E-E pairs in “mvm” subset.

### EnContact accurately predicts enhancer-enhancer contacts

**Figure 2:**
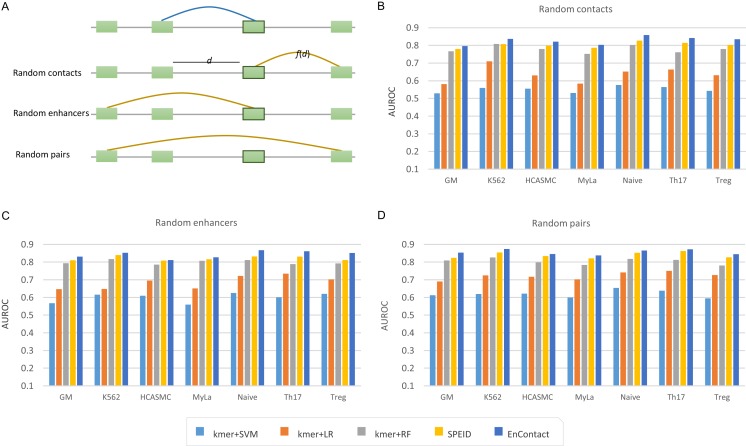
Comparison with baseline methods. (A) Three types of background enhancer-enhancer pairs. Random contacts are generated based on the distance distribution, *f*(*d*), of positive interactions; *d* the distance between two enhancers. Random enhancers are sampled with one end of positive interactions fixed. Random pairs are randomly selected from all possible combinations of any two enhancers. (B–D) Performance of EnContact and other baseline methods across seven cell lines on account of random contacts (B), random enhancers (C), and random pairs (D) as background.

**Figure 3:**
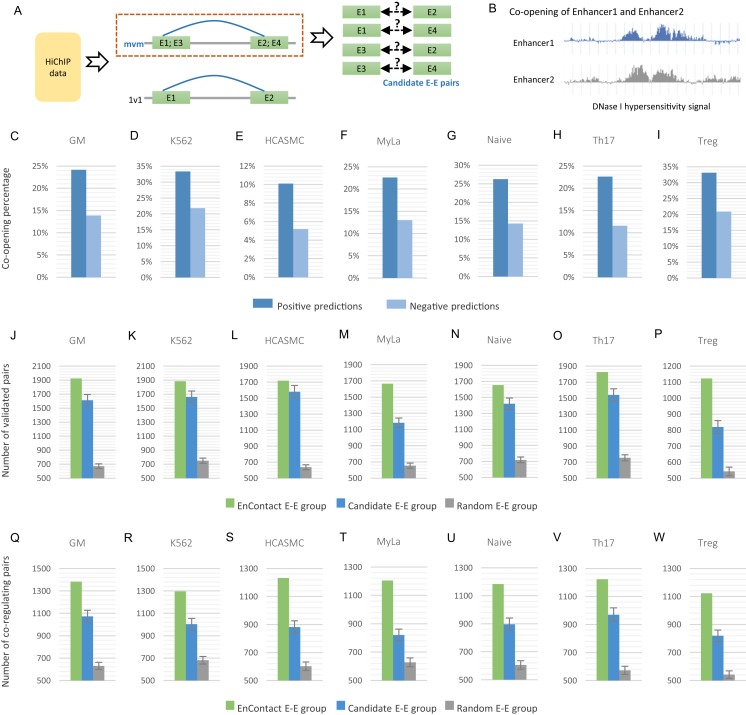
Fine-scale enhancer-enhancer interactions predicted by EnContact. (A) Region-based interactions in HiChIP data are divided into two sets. Interacting regions with more than one enhancer located on each end may result in several candidate enhancer-enhancer interactions. (B) Concept of enhancer co-opening based on DNase I hypersensitivity signal. (C–I) Co-opening percentages of positive predictions and negative predictions across seven cell lines. (J–P) Comparison of validated enhancer-enhancer interactions within E-E pairs predicted by EnContact, E-E pairs derived from candidate interactions, and random E-E pairs. (Q–W) Comparison of three E-E groups (i.e., EnContact E-E group, Candidate E-E group, and Random E-E group) in terms of the number of E-E pairs whose two enhancers regulate the same promoter.

To evaluate the ability of EnContact in predicting E-E interactions, we designed a series of systematical experiments. For each cell line, the unambiguous E-E interactions in “1v1” subset were regarded as positive cases. Meanwhile, we considered three ways to generate negative cases: (1) sampling negative cases based on the distance distribution of positive cases (random contacts; Fig. 2A); (2) randomly sampling negative cases with one end of positive E-E pairs fixed (random enhancers; Fig. 2A); (3) randomly sampling negative cases from all possible combinations of enhancers (random pairs; Fig. 2A). Then, we uniformly divided the union of positive cases and negative cases into 10 groups: one for testing and the others for training. All assessments were conducted on the testing sets.

Next, we trained and evaluated EnContact model in seven cell lines (Table 2). When negative cases were sampled on account of the distance distribution of positive cases, EnContact yields AUROCs of 0.803–0.858 and AUPRCs of 0.773–0.850. Otherwise, when negative cases were sampled based on random enhancers or random pairs, EnContact yields AUROCs of 0.811–0.867 and AUPRCs of 0.800–0.875 (for random enhancers), and AUROCs of 0.838–0.874 and AUPRCs of 0.851–0.891 (for random pairs). This result demonstrates that our EnContact model can achieve decent performance no matter how negative cases were generated. To check whether the shared enhancers between the training set and testing set will bring bias to the model evaluation, we also designed another training and testing sets which involves different groups of enhancers. Specifically, we considered interactions derived from chromosome 1–18 as training set, and interactions derived from other chromosomes as testing set. Our model achieves comparable performance on the new training sets and testing sets (Table S5).

**Table 2:** Model performance of EnContact across seven cell lines.

Cell line	Random contacts		Random enhancers		Random pairs	
	AUROC	AUPRC	AUROC	AUPRC	AUROC	AUPRC
GM	0.806	0.773	0.831	0.830	0.853	0.863
K562	0.837	0.850	0.852	0.826	0.874	0.887
HCASMC	0.821	0.804	0.811	0.815	0.846	0.857
MyLa	0.803	0.792	0.827	0.800	0.838	0.851
Naive	0.858	0.848	0.867	0.875	0.865	0.885
Th17	0.842	0.832	0.861	0.868	0.872	0.891
Treg	0.835	0.828	0.851	0.854	0.845	0.864
Mean	0.829	0.818	0.843	0.838	0.856	0.871

Finally, we compared our EnContact model with four baseline models: SVM (Wu, Lin & Weng, 2004), LR (Hosmer, Lemeshow & Sturdivant, 2013), RF (Liaw & Wiener, 2002), and SPEID model (Singh et al., 2016). In EnContact model and SPEID model, we used a one-hot encoding strategy to convert sequences into a numeric matrix and then extracted features by scanning along the matrix with a convolution layer. For other machine learning models which do not have convolution layers, we need a strategy to extract features from sequences for downstream classification. Here, to derive sequence features, we counted the number of *k*-mer (say, *k* = 6) located in each enhancer and constructed a *k*-mer frequency vector for each enhancer. Then, we concatenated the frequency vector of two enhancers of an E-E pair as the input for baseline models. With the same training sets and testing sets, EnContact achieves a mean AUROC of 0.827, compared with 0.551 of SVM, 0.636 of LR, 0.779 of RF, and 0.802 of SPEID (random contacts; Fig. 2B); a mean AUROC of 0.843, compared with 0.599 of SVM, 0.686 of LR, 0.799 of RF, and 0.821 of SPEID (random enhancers; Fig. 2C); a mean AUROC of 0.856, compared with 0.620 of SVM, 0.722 of LR, 0.804 of RF, and 0.839 of SPEID model (random pairs; Fig. 2D). This shows that our model outperforms other baseline models in all seven cell lines under different backgrounds.

Taken together, the above results show that our EnContact model is capable of extracting useful features from genomic sequences to predict E-E interactions and can achieve much better performance than other traditional methods.

### EnContact captures context-specific features which can be mapped to transcription factors

To explore the biological meaning of features learned by EnContact, we developed a strategy to convert the parameters of the first convolution layer of EnContact into PWM (see “Methods”). Each convolution kernel can be regarded as a pattern recognizer, which recognizes a specific sequence pattern by scanning along the input matrix. Then, we matched the resulting PWM to known motifs and TFs derived from JASPAR database (Mathelier et al., 2016). As a result, we found that EnContact captures different sets of TFs in different cell lines and some of the TFs have been reported to be key TFs in the corresponding cell line (Table 3). For example, IRF4 was identified as a key TF in Th17 cell line by our strategy and was previously reported to be required during the development of inflammatory Th17 cells (Brüstle et al., 2007; Lohoff et al., 2002). In Treg cell line, we matched a convolution kernel to the motif of ETS1, which was previously reported to control the development and function of natural Treg cells (Mouly et al., 2010). The motif analysis shows that EnContact model can capture context-specific sequence patterns and simultaneously convert input sequences into higher-level features. To sum up, EnContact demonstrates the ability to capture known context-specific sequence patterns and provides us an opportunity to explore novel context-specific TFs which have not been identified by experiments yet.

**Table 3:** Key TFs captured by convolution kernels of EnContact.

Cell type	Key TFs
GM	PKNOX1, PKNOX2, FOS, BHLHE40, JUND, USF1, EBF1
K562	EGR1, MGA, BHLHE40, MNT, CREB1, FOXA1, NFYB, USF1
HCASMC	ETS1, IRF2, HSF2, MZF1, HOXB3, JUND, CREB1, FOXO3, POU6F1, NFE2, ZNF263, BATF3, ATF7, NKX6-2
MyLa	ETS1, FLI1, RORA, CREB1, ELK4, NFATC1, ETV3, ZBTB33, REST, ELK3, MLX, NR3C1, SP2, ETV6
Naive	IRF4, PLAG1, IRF2, HSF2, MZF1, HOXB3, MEF2D, JUND, CREB1, EMX1, FOXO3, NFYB, MEF2A, POU6F1
Th17	IRF4, IRF2, FOXP3, FOXP1, TP53, RXRB, SMAD3, NR2C2, POU6F1, CREB1, TFE3, IRF7, PRDM1
Treg	ETS1, NFATC2, MAX, RORA, ELF4, NR2C2, RUNX1, ELF1, RELA, ZBTB33

### EnContact provides a finer mapping of enhancer-enhancer interactions

As discussed in the previous section, we divided E-E interactions derived from HiChIP data into “1v1” subset and “mvm” subset. Chromatin contacts in “mvm” subset are interactions with more than one enhancer in either end or both ends, which therefore can be regarded as a bunch of candidate E-E pairs (Fig. 3A). For a given cell line, once the context-specific model was trained using E-E interactions in “1v1” subset, we can apply this model to predict true interactions from candidate E-E pairs in “mvm” subset.

For each of the seven cell lines, we applied the well-trained EnContact model to identify true interactions from candidate E-E pairs, which can contribute to reducing the false positive rate of E-E pairs for HiChIP data. For each candidate E-E pair, EnContact provides a probability that two enhancers of this pair are interacting with each other. Setting threshold as 0.5, we considered E-E pairs with probabilities larger than the threshold as positive predictions, otherwise as negative predictions. In total, we identified 1,545,180 positive E-E interactions from candidate E-E pairs in seven cell lines.

Next, we compared the co-openness of two enhancers in positive predictions with that in negative predictions. Here, we calculated the co-opening degree of two enhancers based on the consistency of their DNase I hypersensitivity signal (Fig. 3B; see “Methods”). We assumed that if two enhancers are interacting with each other, they should have a larger probability to co-open. Indeed, we found that the percentage of co-opening pairs of positive predictions is larger than that of negative predictions in all seven cell lines (Fig. 3C). This result shows that E-E interactions predicted by EnContact are more likely to be co-opening than other candidate interactions, suggesting that the predicted E-E interactions are more reasonable than those derived from original HiChIP data.

Furthermore, we built a validation dataset using E-E interactions derived from ChIA-PET data of several cell lines to check the consistency between predicted E-E pairs and the validation dataset. We regarded positive predictions inferred by EnContact as “EnContact E-E group.” Then, we constructed a comparison group, named “Candidate E-E group,” by randomly sampling from all candidate pairs with the same sample size as EnContact E-E group. Additionally, we constructed a control group which contains E-E pairs generated based on the distance distribution of positive E-E pairs. The comparison group and control group were generated 1,000 times to remove randomness. In each cell line, we calculated the overlaps between these three E-E groups and the validation dataset. The result shows that in all seven cell lines, EnContact group has the largest overlap with validation interactions, compared with candidate groups and random groups (Fig. 3D). This indicates that EnContact can provide finer mapping E-E interactions than candidate E-E interactions derived from original HiChIP-data.

Finally, considering that E-E interaction may be related with the common promoter that they may co-regulate. We analyzed the E-E interaction based on whether they regulate the same promoter in ChIA-PET data. Specifically, for each E-E group described above (i.e., EnContact E-E group, Candidate E-E group, and Random E-E group), we counted the number of E-E pairs whose two enhancers regulate the same promoter. The result shows that E-E interactions predicted by EnContact model are more likely to co-regulate the same promoters than candidate E-E interactions and random E-E interactions (Fig. 3E). This indicates that EnContact model can identify E-E interactions which are related to the co-regulation of promoters.

Collectively, the above analysis suggests that features learned by EnContact from genomic sequences can be used to predict context-specific E-E interactions.

### Characterization of hub enhancers

**Figure 4:**
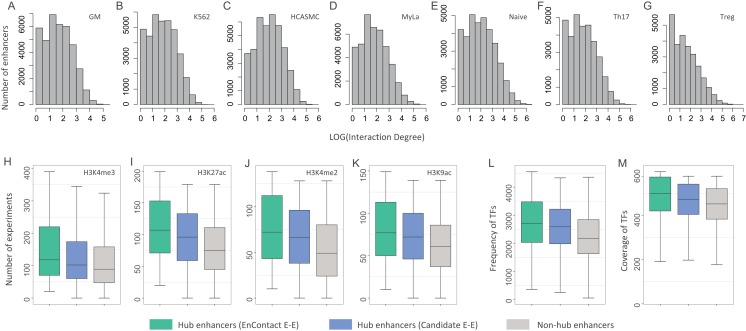
Characterization of hub enhancers. (A–G) Distribution of enhancer interaction degrees in different cell lines. (H–K) Comparison of hub enhancers derived from E-E pairs predicted by EnContact, hub enhancers derived from candidate E-E pairs, and non-hub enhancers in terms of four histone marker H3K4me3 (H), H3K27ac (I), H3K4me2 (J), and H3K9ac (K). (L) Comparison of the number of ChIP-seq experiments where hub enhancers are active. (M) Comparison of the number of TFs included in the experiments where hub enhancers are active. The *y*-axis represents the number of experiments where a promoter is active.

To further discuss the characterization of predicted interactions, we constructed an enhancer-based network using E-E interactions identified by EnContact. We checked the degree distribution of the enhancer network and observed that only a small portion of enhancers have significantly high degrees (Fig. 4A). In the following analysis, we take the data of GM cell line as an example. To check the characterization of enhancers frequently interacting with others, we defined those enhancers with top 10% highest degrees as hub enhancers. For comparison, we prepared hub enhancers of a network constructed using candidate interactions. In addition, we randomly selected non-hub enhancers with the same sample size as another control group. Next, we collected plentiful epigenomic data to explore distinct features of hub enhancers and also to compare the difference of characterization of hub enhancers defined by EnContact interactions, candidate interactions, and non-hub enhancers.

We asked whether hub enhancers are more active across different cell lines than other enhancers. To answer this question, we collected 637 ChIP-seq experiments of 128 cell lines for four key histone markers: H3K4me3, H3K27ac, H3K4me2, and H3K9ac (The ENCODE Project Consortium, 2004). For each hub enhancer, we calculated its activity across different experiments and counted the number of experiments where this enhancer is active. Thus, we derived a distribution of experiment numbers where all hub enhancers defined by EnContact interactions (i.e., E-E pairs predicted by EnContact) are active. Next, we also calculated another two distributions of experiment numbers where hub enhancers defined by candidate interactions (i.e., E-E pairs derived from original HiChIP data) and non-hub enhancers are active. Comparing these three distributions, we observed that hub enhancers defined by EnContact interactions are generally active in significantly more experiments than hub enhancers defined by candidate E-E pairs or non-hub enhancers (Fig. 4B; *P*-values < 2.2 × 10^−16^, one-sided Wilcoxon rank-sum tests). This indicates that hub enhancers are indeed more active across different cell lines. Besides, the comparison result further supports that EnContact is able to extract true E-E interactions from original HiChIP data.

Moreover, we downloaded 4,383 ChIP-seq experiments of 144 cell lines for 579 distinct TFs (The ENCODE Project Consortium, 2004). Similarly, for each hub enhancer, we counted the number of experiments where this enhancer is active and the number of TFs included in these experiments. Then, we compared the number of experiments where hub enhancers define by the three E-E groups are active. As shown in Fig. 4C, hub enhancers defined by EnContact interactions are active in significantly more experiments and covered much more TFs than the other two enhancer groups (*P*-values < 2.2 × 10^−16^, one-sided Wilcoxon rank-sum tests). This again indicates that hub enhancers tend to be active across different cell lines.

In conclusion, EnContact predicts true E-E interactions from original HiChIP data and guides us to identify a class of hub enhancers which tend to be active across different cell lines. Furthermore, the analysis of the characterization of hub enhancers again supports the predicting ability of our EnContact model.

## Discussion

There are several directions worth exploring in future work. First, our sequence-based model can be used to evaluate the influence of nucleotide variants on chromatin interactions. Briefly, for a given SNP located in an enhancer, we can calculate the difference of E-E interacting probability when given reference nucleotide and given variant nucleotide. This difference can be regarded as a quantitative measurement of the biological influence of the given variant. Second, in this work, we only use genomic sequences as input to predict E-E interactions. Since epigenomic features are closely related to three-dimension structure and are complementary to sequence information, we can integrate epigenomic features, which can be derived from ChIP-seq data or ATAC-seq data, with genomic sequences to achieve a better prediction of chromatin contacts among enhancers. Finally, our current model focuses on the prediction of E-E interaction in a context-specific way. With the cooperation of epigenomic signals and sequence information, it is hopeful that an integrative model to predict E-E interactions across cell lines can be developed.

## Conclusions

Chromatin contacts between regulatory elements are of crucial importance for the interpretation of transcriptional regulation and the understanding of disease mechanisms. In the last decade, many computational studies have been developed to improve the resolution of three-dimension genomic data (Whalen, Truty & Pollard, 2016; Zhu et al., 2016; Zhang et al., 2018). However, these methods mainly focused on the interactions between enhancers and promoters, leaving E-E interactions not well explored. In this paper, we designed a deep learning model, EnContact, to predict interactions among enhancers. First, we statistically demonstrated the predicting ability of EnContact using training sets and testing sets derived from HiChIP data of seven cell lines. Then, we compared EnContact with two other machine learning models using *k*-mer features as input. Results show that our model significantly outperforms baseline models. Next, we trained a context-specific model for each cell line and applied the model to predict E-E interactions from original HiChIP data. Finally, we identified hub enhancers from the predicted E-E interactions and observed that hub enhancers tend to be active across cell lines. We summarize that EnContact is capable of predicting E-E interactions using features automatically learned from genomic sequences.

## Supplemental Information

10.7717/peerj.7657/supp-1 Supplemental Information 1 Supplementary Tables.

## Additional Information and Declarations

### Data Availability

The following information was supplied regarding data availability: The EnContact model source code and data is available at https://github.com/liwenran/EnContact.
